# Supplementary material for: Clinical Implementation of an Adapted Infection Risk Screening Tool Following Nurse-Led Haemodialysis Vascular Access Consultation
Source: Healthcare (Basel). 2025 Nov 26;13(23):3058. doi: 10.3390/healthcare13233058 (PMC12691982; doi:10.3390/healthcare13233058)
Supplement: Supplementary file 1 [file healthcare-13-03058-s001.zip › healthcare-3945162-supplementary.pdf]

The STROBE reporting checklist

Title and abstract	Item Description	Location (or reason for not reporting)
1a. Indicate the study's design	Indicate the study's design with a commonly used term in the title or the abstract.	The title and Abstract clearly state that the study is a "retrospective cohort study" (Abstract, lines 6–7; Methods, line 116).
1b. Abstract	Provide in the abstract an informative and balanced summary of what was done and what was found.	The Abstract (lines 1–18) concisely summarises the background, objectives, design, results, and conclusions.
**Introduction**		
2. Background / rationale	Explain the scientific background and rationale for the investigation being reported.	The Introduction (lines 21–91) presents the clinical and epidemiological context, including national and European data on haemodialysis and vascular access, as well as the rationale for investigating infection risk and puncture technique.
3. Objectives	State specific objectives, including any prespecified hypotheses.	The final paragraph of the Introduction (lines 105–114) specifies the study's aims and the prespecified hypothesis that infection risk classification would predict infection-related hospitalisation.
**Methods**		
4. Study design	Present key elements of study design early in the paper.	The study is defined as a retrospective cohort in Methods, Section 2.1 (lines 116–119).
5. Setting	Describe the setting, locations, and relevant dates, including periods of recruitment, exposure, follow-up, and data collection.	The study took place in a Portuguese tertiary hospital. Participants were assessed once during a vascular access (VA) consultation before their first cannulation (lines 116–118); follow-up was passive via electronic health records of hospitalisations at the same institution.
6a. Eligibility criteria	**Cohort study:** Give the eligibility criteria, and the sources and methods of selection of participants. Describe methods of follow-up. **Case-control study:** Give the eligibility criteria, and the sources and methods of case ascertainment and control selection. Give the rationale for the choice of cases and controls. **Cross-sectional study:** Give the eligibility criteria, and the sources and methods of selection of participants.	All adults (≥18 years) with a matured AVF referred for consultation between 1 January 2022 and 31 December 2024 were eligible (lines 122–126). Exclusion: AV grafts. Follow-up lasted 12 months per participant.
6b. Matching criteria	**Cohort study:** For matched studies, give matching criteria and number of exposed and unexposed. **Case-control study:** For matched studies, give matching criteria and the number of controls per case.	Not applicable
7. Variables	Clearly define all outcomes, exposures, predictors, potential confounders, and effect modifiers. Give diagnostic criteria, if applicable.	Exposure: binary infection-risk classification based on the adapted BRS screening tool.<br>Outcomes: (i) any infection-related hospitalisation within 12 months; (ii) number of such admissions.<br>Covariates included age, sex, BMI, diabetes, CKD stage, vascular access history and modality at consultation (lines 127–177).
8. Data sources /	For each variable of interest give sources of data and details of methods of	All exposures and covariates were assessed during the

measurement	assessment (measurement). Describe comparability of assessment methods if there is more than one group.	consultation using standardised procedures, clinical records, and point-of-care ultrasound (Section 2.5). Hospitalisation diagnoses were retrieved from hospital records and validated (lines 165–182).
9. Bias	Describe any efforts to address potential sources of bias.	Potential biases are discussed in Section 2.5 and again in the Strengths and Limitations (p. 11). Bias mitigation strategies included prospective exposure assessment, standardised outcome validation, and adjusted multivariable models.
10. Study size	Explain how the study size was arrived at.	All eligible individuals during the three-year period were included (n = 404); no sample size calculation was performed due to the retrospective nature (line 190).
11. Quantitative variables	Explain how quantitative variables were handled in the analyses. If applicable, describe which groupings were chosen, and why.	Variables such as age and AVF vintage were analysed as continuous or categorised based on clinical cut-points. Rationale is stated in the Statistical Analysis subsection (lines 190–197).
12a. Statistical methods	Describe all statistical methods, including those used to control for confounding.	Logistic regression was used for binary outcomes; negative binomial regression for count outcomes due to overdispersion (lines 198–227).
12b. Statistical methods – subgroups and interactions	Describe any methods used to examine subgroups and interactions.	No formal subgroup or interaction analyses were performed.
12c. Statistical methods – missing data	Explain how missing data were addressed.	Participants with missing core variables were excluded. Hospital outcome data were complete (line 182).
12di. Statistical methods – loss to follow-up	**Cohort study:** If applicable, describe how loss to follow-up was addressed.	Not applicable. Outcome data were retrieved passively for all participants via hospital records.
12dii. Statistical methods – matching cases and controls	**Case-control study:** If applicable, explain how matching of cases and controls was addressed.	Not applicable.
12diii. Statistical methods – sampling strategy	**Cross-sectional study:** If applicable, describe analytical methods taking account of sampling strategy.	Not applicable.
12e. Statistical methods – sensitivity analyses	Describe any sensitivity analyses.	None were conducted.
**Results**		
13a. Participant numbers	Report the numbers of individuals at each stage of the study—e.g., numbers potentially eligible, examined for eligibility, confirmed eligible, included in the study, completing follow-up, and analysed; Consider use of a flow diagram.	The Results section (line 232) and Table 4 present the total cohort (n = 404) and event distribution.
13b. Participants – non-participation	Give reasons for non-participation at each stage.	Not applicable. All eligible individuals were included.
13c. Participants – flow diagram	Consider use of a flow diagram.	Not provided; cohort construction is described textually (lines 116–126).
14a. Descriptive data – participant characteristics	Give characteristics of study participants (e.g., demographic, clinical, social) and information on exposures and potential confounders. Present the information in a table.	Table 1 and Results (lines 232–248) present demographic and clinical characteristics.
14b. Descriptive data –	Indicate the number of participants with missing data for each variable of	Cases with incomplete data were excluded; no imputation

missing data	interest.	performed (line 182).
14c. Descriptive data – follow-up time	**Cohort study:** Summarise follow-up time—e.g., average and total amount.	A fixed 12-month follow-up was applied to all participants (line 153).
15. Outcome data	**Cohort study:** Report numbers of outcome events or summary measures over time. **Case-control study:** Report numbers in each exposure category, or summary measures of exposure. **Cross-sectional study:** Report numbers of outcome events or summary measures.	Infection-related hospitalisations are detailed in Tables 2 and 3 (lines 256–263).
16a. Main results	Give unadjusted estimates and, if applicable, confounder-adjusted estimates and their precision (e.g., 95% confidence intervals). Make clear which confounders were adjusted for and why they were included.	Adjusted odds ratios and incidence rate ratios are reported in Table 4 (page 9).
16b. Main results – category boundaries	Report category boundaries when continuous variables were categorised.	Cut-points and categorisation are described in Section 2.7 (lines 195–197).
16c. Main results – risk	If relevant, consider translating estimates of relative risk into absolute risk for a meaningful time period.	Percentages of hospitalised individuals are presented (Table 3, line 262).
17. Other analyses	Report other analyses done—e.g., analyses of subgroups and interactions, and sensitivity analyses.	Negative binomial regression was applied to explore infection admission counts (line 216 onward).
**Discussion**		
18. Key results	Summarise key results with reference to study objectives.	Summarised in the opening of the Discussion (lines 303–315), contextualising the findings.
19. Limitations	Discuss limitations of the study, considering sources of potential bias or imprecision. Discuss both direction and magnitude of any potential bias.	Discussed explicitly in the Strengths and Limitations section (lines 372–391), including potential biases and generalisability.
20. Interpretation	Give a cautious overall interpretation considering objectives, limitations, multiplicity of analyses, results from similar studies, and other relevant evidence.	A balanced interpretation is offered, integrating prior literature and clinical relevance (lines 303–370).
21. Generalisability	Discuss the generalisability (external validity) of the study results.	Addressed in lines 377–391, reflecting on the representativeness of the setting and practice model.
**Other information**		
22. Funding	Give the source of funding and the role of the funders for the present study and, if applicable, for the original study on which the present article is based.	Declared in the final section: "No external funding was received" (line 399).
